# Prediction of Scar Size in Rats Six Months after Burns Based on Early Post-injury Polarization-Sensitive Optical Frequency Domain Imaging

**DOI:** 10.3389/fphys.2017.00967

**Published:** 2017-12-01

**Authors:** Eli Kravez, Martin Villiger, Brett Bouma, Martin Yarmush, Zohar Yakhini, Alexander Golberg

**Affiliations:** ^1^School of Computer Science, Interdisciplinary Center Herzliya, Herzliya, Israel; ^2^Wellman Center for Photomedicine, Massachusetts General Hospital, Harvard Medical School, Boston, MA, United States; ^3^Institute of Medical Engineering and Science, Massachusetts Institute of Technology, Cambridge, MA, United States; ^4^Department of Surgery, Center for Engineering in Medicine, Massachusetts General Hospital, Shriners Burns Hospital for Children, Harvard Medical School, Boston, MA, United States; ^5^Department of Biomedical Engineering, Rutgers University, Piscataway, NJ, United States; ^6^Porter School of Environmental Studies, Tel Aviv University, Tel Aviv, Israel

**Keywords:** scars size prediction, burn injury, wound healing diagnosis, optical coherence tomography, skin imaging

## Abstract

Hypertrophic scars remain a major clinical problem in the rehabilitation of burn survivors and lead to physical, aesthetic, functional, psychological, and social stresses. Prediction of healing outcome and scar formation is critical for deciding on the best treatment plan. Both subjective and objective scales have been devised to assess scar severity. Whereas scales of the first type preclude cross-comparison between observers, those of the second type are based on imaging modalities that either lack the ability to image individual layers of the scar or only provide very limited fields of view. To overcome these deficiencies, this work aimed at developing a predictive model of scar formation based on polarization sensitive optical frequency domain imaging (PS-OFDI), which offers comprehensive subsurface imaging. We report on a linear regression model that predicts the size of a scar 6 months after third-degree burn injuries in rats based on early post-injury PS-OFDI and measurements of scar area. When predicting the scar area at month 6 based on the homogeneity and the degree of polarization (DOP), which are signatures derived from the PS-OFDI signal, together with the scar area measured at months 2 and 3, we achieved predictions with a Pearson coefficient of 0.57 (*p* < 10^−4^) and a Spearman coefficient of 0.66 (*p* < 10^−5^), which were significant in comparison to prediction models trained on randomly shuffled data. As the model in this study was developed on the rat burn model, the methodology can be used in larger studies that are more relevant to humans; however, the actual model inferred herein is not translatable. Nevertheless, our analysis and modeling methodology can be extended to perform larger wound healing studies in different contexts. This study opens new possibilities for quantitative and objective assessment of scar severity that could help to determine the optimal course of therapy.

## Introduction

Burns is a global public health problem, accounting for an estimated 265,000 deaths annually (WHO, 2016). A review of literature published since 1965 reported a 32–72% prevalence rate of hypertrophic scarring in patients suffering from burn injuries (Lawrence et al., 2012). Although tremendous progress has been achieved in the last decades in saving lives after burn injuries, hypertrophic scars remain a major clinical problem in the rehabilitation of burn survivors, leading to physical, aesthetic, functional, psychological, and social stresses (Lawrence and Fauerbach, 2003; Aarabi et al., 2007; Baillie et al., 2014). Moreover, molecular mechanisms behind scar formation are not completely understood and it remains challenging to provide early prediction of the long term scar outcome (Tziotzios et al., 2012; Koppenol et al., 2017).

Prediction of scarring outcome at the early time points after injury is important for the decisions about burn wound management and treatment planning and assessment (Sheridan, 2012; Koppenol et al., 2017). Therefore, a range of subjective and objective scales has been devised to assist the caregiver in the decision making (Heimbach et al., 1984). The subjective scales, such as The Vancouver Scar Scale (VSS), Manchester Scar Scale (MSS), Patient and Observer Scar Assessment Scale (POSAS), Visual Analog Scale (VAS), and Stony Brook Scar Evaluation Scale (SBSES), consider factors such as scar height or thickness, pliability, surface area, texture, pigmentation, and vascularity (Nedelec et al., 2000; Fearmonti et al., 2010). However, they are dependent on the observer and are difficult to compare. In search of more objective diagnostic burn criteria, a wide spectrum of methods has been explored: biopsy and histology (Sheridan, 2012), fluorescent imaging (Sheridan et al., 2015), near-infrared light spectroscopy (Cross et al., 2007), confocal, and multiphoton microscopy (Chen et al., 2011), laser Doppler techniques (Jaskille et al., 2010), and non-contact high-frequency ultrasonography (Lin et al., 2011), as well as thermography (Liddington and Shakespeare, 1996). To date, these strategies fall into one of two categories: (1) they lack the ability to image individual layers of the wound and provide an accumulated bulk signal, making the diagnosis of burn depth unreliable, and (2) although providing high spatial resolution and depth-sectioning, the limited field of view and long acquisition times make them impractical in a clinical setting. In contrast, Optical Frequency Domain Imaging (OFDI) and related implementations of optical coherence tomography (OCT) provide depth-resolved images of the tissue architecture and functional vasculature with an interesting trade off of the field of view, spatial resolution, and imaging speed, that can help to overcome the barriers encountered by alternative modalities (Park et al., 2001; Kim et al., 2012; Villiger et al., 2013a,b).

OFDI is an optical imaging modality that captures micrometer-resolution, three-dimensional images of the subsurface microstructure of biological tissues. Polarization Sensitive (PS) OFDI further improves structural imaging by providing insight into the polarization properties of the tissue by measuring the polarization state of the light backscattered by the tissue. The polarization state of light is altered by propagating through a medium that exhibits birefringence. Quantifying the rate of this change with depth provides a measure of tissue birefringence. The majority of the extracellular matrix of skin consists of collagen, which is a prominent source of birefringence. In skin undergoing thermal injury, the collagen proteins denature, in the process of which they lose their birefringence. Hence, burns are characterized by a lower birefringence, a feature which previously has been used to assess burn depth (Park et al., 2001; Kim et al., 2012). In addition to birefringence, PS-OFDI can also assess depolarization, which corresponds to a randomization of the measured polarization states. This randomization is expressed by the Degree of Polarization (DOP), ranging from 0 for completely randomized polarization states to 1 for perfectly aligned polarization states. Collagen fibers that are uniformly arranged result in deterministic polarization states that vary along depth due to tissue birefringence (Lo et al., 2016) but are locally uniform and result in high DOP. In contrast, collagen fibers that are disorganized on a size scale smaller than the focal volume of the probing beam result in a randomization of the measured polarization states and a reduced DOP (Lo et al., 2016).

We previously have developed reconstruction strategies that mitigate artifacts resulting from polarization sensitive measurements through fiber-based imaging systems and provide maps of tissue birefringence and DOP in animal models (Villiger et al., 2013a,b). We have also shown that PS-OFDI provides valuable insights into the structural remodeling taking place during scar formation in a mechanical tension induced HTS model in rats (Lo et al., 2016). Compared to normal skin with heterogeneous birefringence and low DOP, HTS was characterized by an initially low birefringence, which increased as collagen fibers remodeled, and a persistently high DOP (Lo et al., 2016). In additional work, we showed that PS-OFDI signature could differentiate between third-degree burn scars treated with different therapy plans of partially irreversible electroporation (Golberg et al., 2016).

The goal of the present work was to develop a linear regression model that predicts the size of the scar 6 months after a third-degree burn in rats based on PS-OFDI imaging at early time points. Linear regression models are the simplest prediction models for quantitative inference [see ref (Rice, 2010) as well as (Stanton, 2001) for a historical perspective]. Such models are used in diverse contexts including biology and medical science (Motulsky and Christopoulos, 2002) as well as economics and social science (Greene, 2011). Accurately predicting the healing outcome of burn injuries based on non-invasive imaging at early time points would be a crucial diagnostic capability that could open new options for management and care of burn patients.

## Materials and methods

### Animals

Female Sprague-Dawley rats (~250 g, *N* = 18, 6-week-old) were purchased in Charles River Laboratories (Wilmington, MA). The animals were housed in cages with access to food and water *ad libitum* and were maintained on a 12-h light/dark cycle in a temperature-controlled room. All animal procedures were approved by the Institutional Animal Care and Use Committee (IACUC) of the Massachusetts General Hospital. All procedures were in accordance with the guidelines of the National Research Council. The animals were treated humanely.

### Scar models

For statistical prediction of the resulting scar size, a dataset with a range of different scar sizes is needed. We used the data from our previous work where we showed the ability of partially irreversible electroporation (pIRE) to reduce the size of scars in rats, 6 months after injury (Golberg et al., 2016). Third degree burns were treated at various time points after injury with different pulsed electric field parameters, resulting in a range of scar sizes 6 months after the initial injury, as reported in Golberg et al. (2016). In brief, animals were anesthetized with isoflurane and their fur was clipped along the dorsal surfaces. Burns were incurred by pressing the end of a pre-heated (≥95°C) brass block against the rat's dorsum for 10 s, resulting in a non-lethal, full-thickness, third-degree burn, measuring ~1 cm^2^, which is 0.25% of the total body surface area (TBSA; Golberg et al., 2016). Four burn injuries were performed on each animal at sites separated by 2 cm along the head to tail axis, accounting for 1% TBSA of total burn area. The depth of the burn was evaluated histologicaly in 9 animals at time 0, 12 h and 1 week after the injury (*n* = 3 animals per time point; Golberg et al., 2016). One burn served as control and three burns with treated with pIRE using contact electrodes with a surface area of 1 cm^2^, separated by a 2 mm gap (Golberg et al., 2016). Square pulses of 70 μs duration at a 3 Hz repetition rate were delivered using a BTX 830 pulse generator (Harvard Apparatus Inc., Holliston, MA; Golberg et al., 2016). Voltage, number of pulses, and treatment frequency very varied between different groups of animals and are described in Table S1 (Golberg et al., 2016). In total we analyzed 3 replicate wounds per treatment condition for each of the 9 different treatment conditions.

### Polarization-sensitive optical frequency domain imaging

PS-OCT was performed as reported in detail previously (Villiger et al., 2013a; Lo et al., 2016), at 1, 2, 3, 5, and 6 months after the burn injury. The system operated with a wavelength-swept laser source at an A-line rate of 54 kHz and a center wavelength of 1,320 nm, achieving an axial resolution of 9.4 μm in tissue. We scanned rectangular surface areas of 10 × 5 mm, consisting of 2,048 A-lines/image × 256 images, with a focused beam featuring a lateral resolution of 15 μm. The lesions were covered with a thin layer of ultrasound-gel as an immersion liquid and apposed against a glass slide to center the superficial layers in focus. Two to three volumes were acquired for each lesion and time point, and the volume that aligned most accurately with the lesion was selected for further analysis. For PS-OCT, the polarization state of the light directed to the sample was alternated between linear and circular polarization between adjacent A-lines, and the signal was detected with a polarization diverse receiver.

The data were reconstructed with spectral binning (Villiger et al., 2013a), using 1/5th of the spectral bandwidth, a lateral Gaussian filter with a full width at half maximum (FWHM) equal to 12 adjacent A-lines, and an axial offset of 48 μm to derive depth-resolved tissue birefringence. Tissue birefringence was expressed in deg/μm, corresponding to the amount of retardation per sample path. The DOP was evaluated independently for each spectral bin and input polarization state over the same lateral Gaussian kernel, and then averaged:

(1) $$\begin{array}{r} DOP=\frac{1}{2N}\sum_{p=1}^{2}\sum_{n=1}^{N}\frac{\sqrt{Q_{p,n}^{2}\text{+}U_{p,n}^{2}\text{+}V_{p,n}^{2}}}{I_{p,n}} \end{array}$$

where *Q, U, V*, and *I* are the spatially averaged components of the Stokes vector, *n* denotes the spectral bin and *p* the input polarization state. DOP expresses the randomness of the measured polarization states and scales from 0 (completely random) to unity (uniform). Close to the surface, the polarization states are usually well maintained, resulting in a DOP close to unity. As the light propagates deeper and depending on the depolarizing properties of the tissue, the light gets increasingly depolarized, resulting in lower DOP values. The structural intensity tomograms are displayed in logarithmic scale as gray scale images. Birefringence was mapped from 0 to 1.2 deg/μm with an isoluminant color map (Geissbuehler and Lasser, 2013) and overlayed with the gray-scale intensity image. DOP is scaled from 0.5 to 1 and is rendered in the same color map.

For quantitative analysis of the polarization properties, we defined a cylindrical region of interest with a diameter of 1 mm centered on the lesion at each time point and extending from the epidermis to the subcutaneous fat. We found that both mature scars and normal skin resulted in high mean birefringence values.

In scars, the birefringence is very uniform and accompanied by a high DOP. In contrast, the mesh-like arrangement of collagen fibrils in the normal skin results in a spot-like, heterogeneous appearance of birefringence in normal skin, paired with low DOP. Hence, we used a measure of homogeneity of the birefringence to best capture the scar status. Homogeneity was evaluated with the “graycomatrix” function, available in the image processing toolbox in Matlab (MathWorks, Natick, MA, USA). It was computed with an offset of 5 pixels in the axial direction, and dividing the birefringence into 12 levels ranging from 0 to 1 deg/μm over the entire cylindrical region of interest. Homogeneity results in a value from 0 (not homogeneous) to 1 (very homogeneous). As a second polarization metric, we computed the average slope of the DOP (DOPSlope). The DOP values were averaged at each depth across the tissue cylinder and then fit with a straight line to express its downward slope per millimeter.

### Quantification of scar area

Scar surface areas were quantified from digital images, captured at each time point, with ImageJ software (Schneider et al., 2012). All scar edges were traced manually and the area was quantified using a calibrated internal length standard for each image.

### Linear regression model to predict scar area

The data set consists of 36 data points (wounds): 9 animals with 4 lesions each (1 untreated burn and 3 burns treated with pIRE). For each wound three features were measured each month (Scar_Area, Homogeneity, and DOPSlope).

We performed linear regression using two types of data vectors as features for predicting the scar size at 6 months after the burn injury:

- 3-dimensional data vectors, including Scar_Area, Homogeneity, and DOPSlope for only a single month from months 1 to 3.
- 6-dimensional data vectors, including the same features, taken from a pair of months from months 1 to 3.
- 9-dimensional data vectors, including the same features, taken from months 1, 2, and 3.

We first transformed all feature values into z-score explanatory variables:

(2) $$\begin{array}{r} z=\;\frac{\left(x-\mu \right)}{\sigma } \end{array}$$

Where μ and σ are the mean and standard deviation for the feature, across the 36 samples, and x is the measured feature value.

We used leave-one-out cross-validation (LOOCV) as follows—we built a linear regression based on 35 samples and predicted the target value (scar size at month 6) for the hidden data point. This process was repeated 36 times (each sample left out once). The LOOCV process results in 36 predictions for the scar area at month 6.

We then calculated the Pearson and Spearman correlations (Rice, 2010) between the vector of predicted values and the vector of actual measured scar sizes. The Root Mean Square Error (RMSE) of the prediction model was also determined as follows:

(3) $$\begin{array}{r} RMSE=\;\sqrt{\frac{1}{n}\overset{i}{\underset{1}{\sum }}{\left(x_{i}-\;{\hat{x}}_{i}\right)}^{2}} \end{array}$$

where *x*_*i*_ is the measured value for the scar size, ${\hat{x}}_{i}$ is the value predicted by a given model, and n is the number of samples.

We chose to use several approaches to measure the prediction quality as they represent different aspects that may be of interest. Pearson correlation provides an estimate of how the predicted values are linearly associated with the measured ones. Spearman correlation provides an estimate of how monotonic the predicted values are compared to the measured ones. That is—how predictive the early post-injury measurements are in being able to answer questions like—will Scar A be larger than Scar B at month 6? RMSE provides an intuitive quantitative estimate of the absolute error.

Amongst these approaches to evaluating performance Spearman correlation is the most robust one, insensitive to the variation that can be calibrated in practice and to the statistical model selection.

### Random control

As a random control, we shuffled the measured values at month 6 and performed the same LOOCV analysis for the shuffled data. This way we disconnect the relationship between the measurements at month 1–3 and the corresponding 6th^−^month scar size. This yields datasets that are identical to the original ones but with shuffled 6th-month scar size. The shuffling and LOOCV analysis were repeated 1,000 times. For each such shuffled instance, we compute the Pearson and Spearman of the predicted Scar_Area at 6th month to the input (shuffled) Scar_Area at 6th month. Thereby we obtain an empirical *p*-value for the observed performance of predicting the actual real data and can rule out overfitting. We performed this analysis for all model types described above.

## Results

Our hypothesis is that PS-OFDI signatures measured at the early time points after the burn injury allow predicting the final wound healing outcome. To test this hypothesis, we simplified this complex process and defined the scar size (area in mm^2^) at 6 months after the injury as the healing outcome and modeled it in response to the PS-OFDI signatures and the scar size at early time points. We used the homogeneity of the birefringence (Hom) and the slope of the DOP (DOPSlope) as signatures derived from the PS-OFDI measurements. These parameters were evaluated in a cylindrical region of interest in the center of each lesion (2–3 volumes per lesion), extending from the epidermis to the subcutaneous fat. Together with the scar size, as measured from digital photographs, this leads to the following model:

(4) $$\begin{array}{l} Scar\_Area_{\left(6m\right)}=\beta_{0}\;{+\sum_{i=1}^{K}\alpha_{i}Scar\_Area_{i}\;+\gamma_{i}Hom_{i}}^{\text{​}}\\ \;+\delta_{i}DOPSlope_{i}, \end{array}$$

where Scar_Area is the surface area of the burn scar (mm^2^), Hom, and DOP_Slope are the OFDI derived homogeneity and DOP slope (Golberg et al., 2016), *i* indicates the time point in months after the burn injury, K is the set of employed time points (1, 2, or 3 months after the injury with all combinations), β_0_ is the intercept, and α, γ, and δ are scalar coefficients obtained by linear regression.

**Figure 1:**
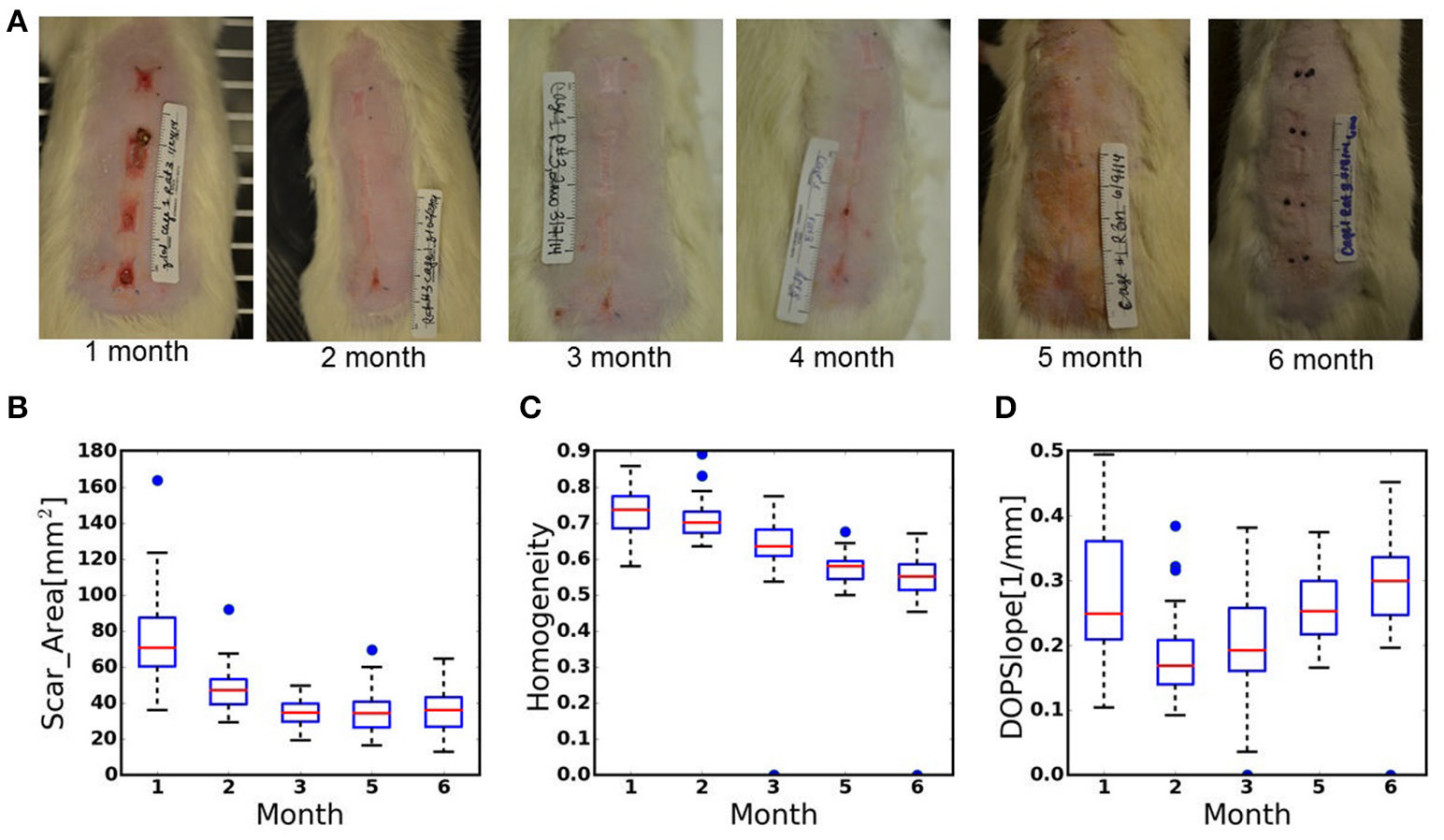
**(A)** Digital images of scar remodeling over time up to 6 months after third-degree burn injury. **(B–D)** Box-plots of the data recorded from the scars during 6 months of healing. **(B)**. Scar_Area, **(C)**. Homogeneity of birefringence, **(D)**. The slope of the DOP. Plot annotations were defined as follows. Boxes: the main body of the boxplot showing the quartiles and the median's confidence intervals if enabled. Medians: horizontal lines at the median of each box. Whiskers: the vertical lines extending to the most extreme, non-outlier data points. Caps: the horizontal lines at the ends of the whiskers (*n* = 36 wounds, 9 animals).

**Figure 2:**
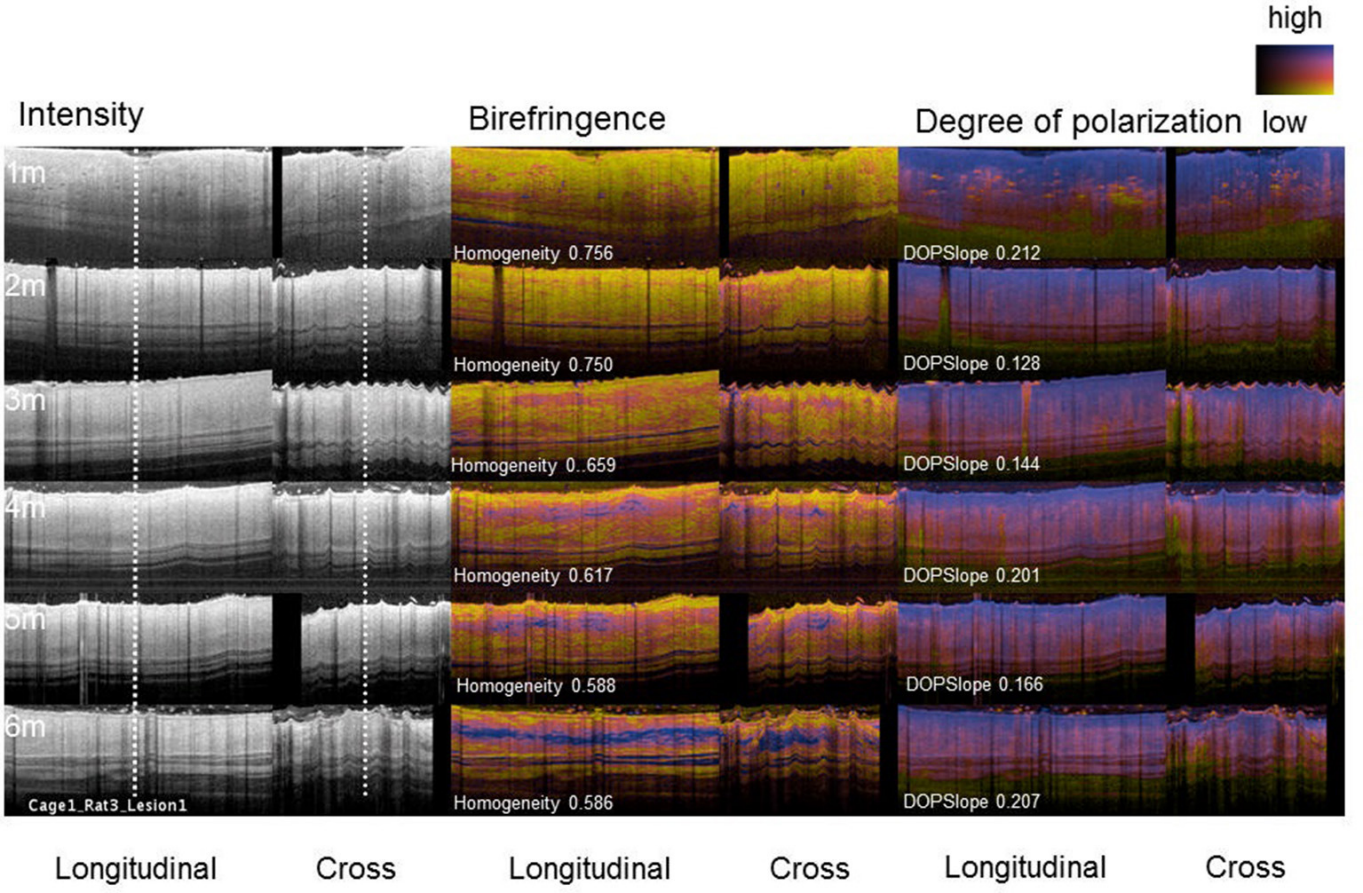
PS-OFDI images of a developing scar following third degree burn injury in the dorsal skin of a rat. Longitudinal and cross-sectional views of the healing burn wounds at several time points in the same animal (*n* = 36 wounds, 9 animals, 2–3 volumes acquired from each wound).

For the construction of the multivariable regression model, we used data, generated in our previous study, where we treated third-degree burns in rats with various pIRE therapy parameters to investigate their impact on the resulting scar size (Golberg et al., 2016). These experiments generated a data set of 36 individual scars of various sizes on 9 animals (one untreated scar per animal and three pIRE treated scars with three biological repeats), measured longitudinally up to 6 months after the burn injury (Figure 1A). A total of 216 measurements for Scar_Area, Hom, and DOPSlope as shown in Table 1 and Figures 1B–D, 2 were used. We used Scar_Area, Hom, and DOPSlope from various combinations of measurements at months 1–3 to predict the scar size in the 6th month.

**Table 1:** Measured parameters of third-degree burn scars in rats with/without pIRE treatment.

	**Scar_Area (mm**^**2**^**)**					**Hom**					**DOPSlope (1/mm)**				
**Month**	**1**	**2**	**3**	**5**	**6**	**1**	**2**	**3**	**5**	**6**	**1**	**2**	**3**	**5**	**6**
Mean	76.13	48.07	33.77	34.72	36.09	0.734	0.7	0.593	0.573	0.539	0.281	0.185	0.195	0.256	0.295
Std	25.22	12.36	7.65	11.01	12.79	0.071	0.055	0.189	0.04	0.1	0.1	0.067	0.09	0.054	0.087
Min	36.068	29.448	19.3	16.519	12.737	0.58	0.635	0.0	0.5	0.0	0.104	0.092	0.0	0.166	0.0
25%	60.2	39.13	29.74	26.61	26.847	0.685	0.672	0.6	0.544	0.514	0.209	0.14	0.16	0.217	0.246
50%	70.69	46.97	34.51	34.29	35.91	0.736	0.701	0.634	0.579	0.552	0.249	0.168	0.192	0.253	0.299
75%	87.46	53.26	39.68	40.6	43.32	0.774	0.731	0.681	0.594	0.585	0.361	0.2	0.257	0.299	0.335
Max	163.736	92.247	49.528	69.56	64.71	0.858	0.892	0.775	0.676	0.671	0.495	0.385	0.382	0.375	0.452

*Dataset is based on individual 36 scars, measured over a period of 6 months after initial burn injury, as reported in Golberg et al. (2016). Shown data is an average of 9 animals (4 wounds per animal). OFDI data for each wound includes an average from 2 to 3 acquired volumes*.

**Figure 3:**
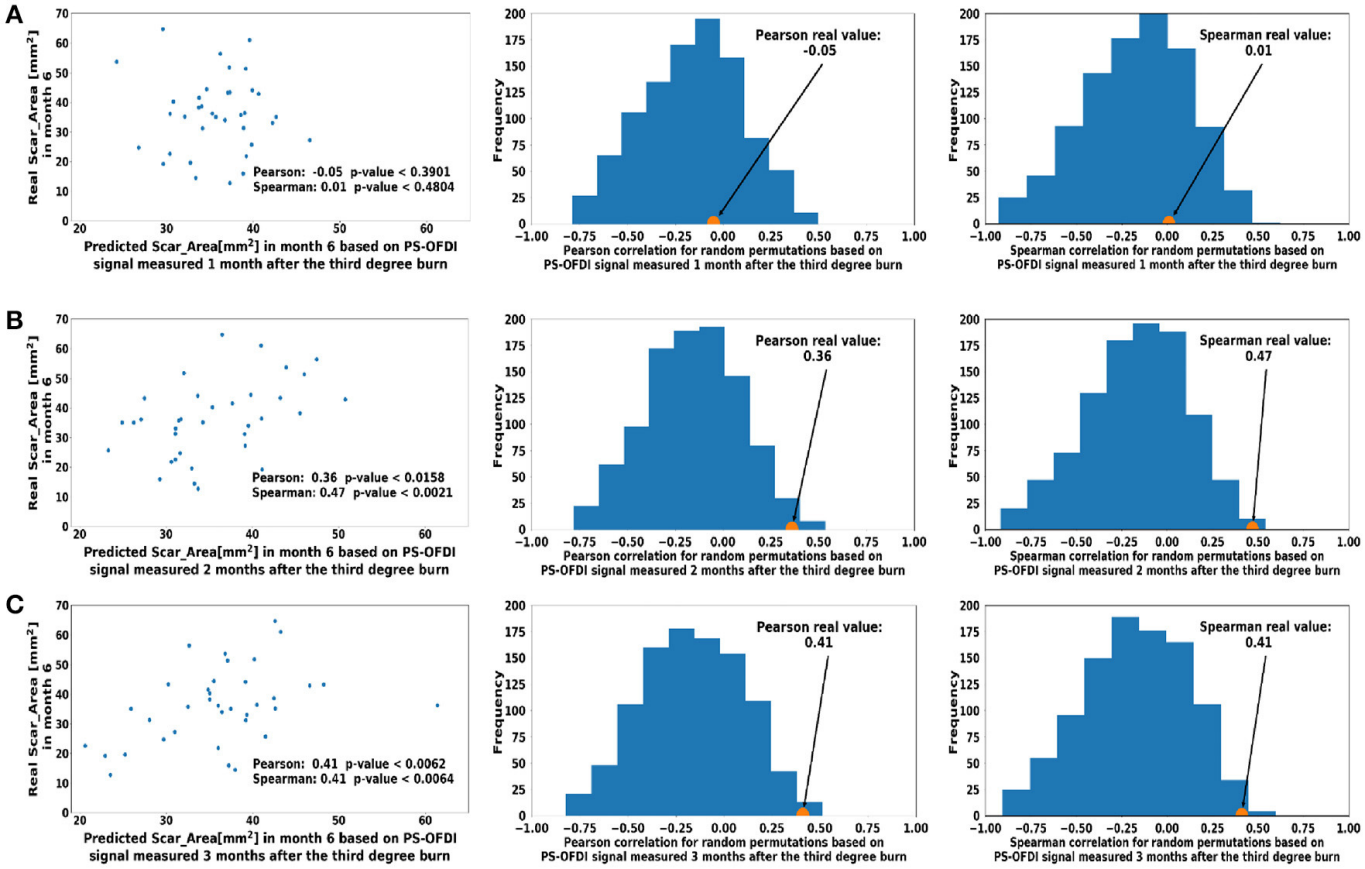
Predicted scar area at month 6 based on measurements taken at a single time point **(A)**. Month 1 **(B)**. Month 2 **(C)**. Month 3 after third-degree burn injury. The control histograms of the correlation coefficients corresponding to randomly shuffled measurements appear on the right-hand side of each panel.

First, we attempted to predict the Scar_Area at month 6 based on the Scar_Area, Hom, and DOPSlope measured only at the single time point of month 1, 2, or 3 after the injury (pIRE therapy was ongoing during this period; Golberg et al., 2016). The predictions based on the measurements 1 month after the burn injury were not significantly correlated to the actual measured scar size (Pearson coefficient −0.05, *p* < 0.39 and Spearman coefficient 0.01, *p* < 0.48, Figure 3A). The predictions based on the measurements performed 2 months after the burn injury were more significantly correlated (Pearson coefficient 0.36, *p* < 0.015 and Spearman coefficient 0.47, *p* < 0.002, Figure 3B). The predictions based on the measurements taken 3 months after the burn injury were also significant (Pearson coefficient 0.41, *p* < 0.006 and Spearman coefficient 0.41, *p* < 0.006, Figure 3C).

**Figure 4:**
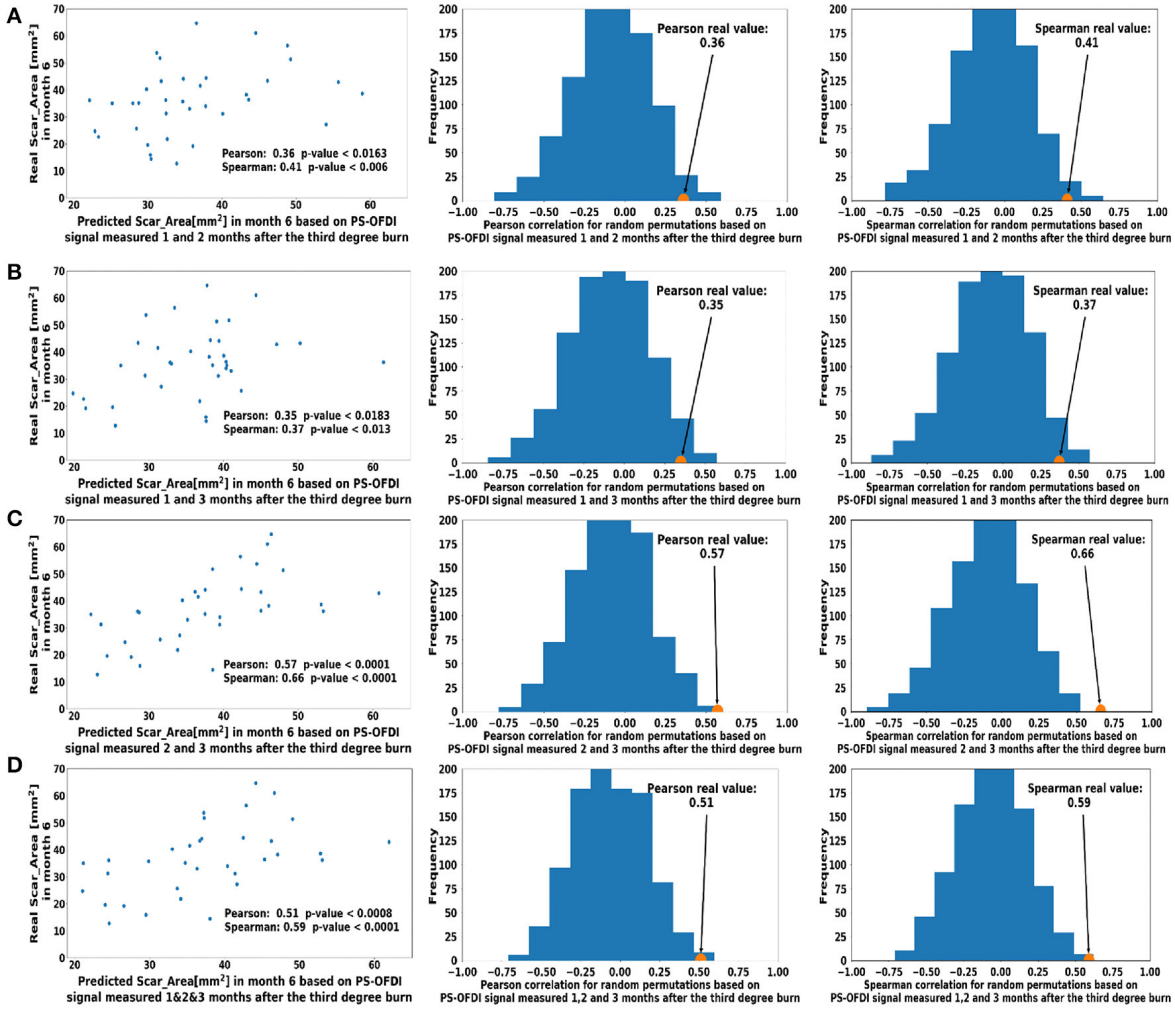
Predicted scar area at month 6 based on multiple measurements taken at **(A)**. Month 1 and Month 2 **(B)**. Month 2 and Month 3 **(C)**. Month 1 and 3 **(D)**. Month 1, 2, and 3 after the third-degree burn injury. The control histograms of the correlation coefficients corresponding to randomly shuffled measurements appear on the right-hand side of each panel.

Next, we extended the model to predict the Scar_Area at month 6 based on the Scar_Area, Hom, and DOPSlope measured at 2 or 3-time points 1, 2, and 3 months after the burn injury. Predictions of the scar areas at month 6 were the most accurate when based on the combined measurements of months 2 and 3 (Pearson coefficient 0.57, *p* < 10^−4^ and Spearman coefficient 0.66, *p* < 10^−5^, Figure 4B). The prediction based on measurements at all 3-time points, months 1, 2 and 3 followed closely in performance (Pearson coefficient 0.51, *p* < 7·10^−4^ and Spearman coefficient 0.59, *p* < 8·10^−5^, Figure 4D). Models of the scar area based on the combined measurements of months 1 and 2 (Figure 4A) or 1 and 3 (Figure 4C) were less significant and resulted in a Pearson coefficient of 0.36, *p* < 0.016 and Spearman coefficient of 0.41, *p* < 0.006 for months 1 and 2, and a Pearson coefficient of 0.35, *p* < 0.018 and Spearman coefficient of 0.37, *p* < 0.013 for months 1 and 3, respectively. The summary of the model comparison appears in Table 2. The model coefficients are Figure 4 reported in Tables S2–S8.

**Table 2:** Summary of the linear regression model for predicting the scar area 6 months after third-degree burn injury based on early time point measurements with PS-OFDI.

**PS-OFDI measurements time points**	**Pearson coefficient (*p*-value)**	**Spearman coefficient (*p*-value)**	**RMSE**
Month 1	−0.04 (0.39)	0.008(0.48)	13.63
Month 2	0.358(0.015)	0.465(0.002)	12.36
Month 3	0.412(0.006)	0.41(0.006)	11.7
Month 1&2	0.356(0.016)	0.413(0.006)	12.6
Month 1&3	0.349(0.018)	0.37(0.012)	12.4
Month 2&3	0.569(0.0001)	0.658(<0.0001)	10.81
Month 1&2&3	0.508(0.0007)	0.587(<0.0001)	11.61

## Discussion

Our long-term goal is to develop a metric based on PS-OFDI signatures to assess scar severity and predict the healing potential of burn wounds. Working toward this goal, in the current study, we tracked the remodeling of healing burns in rats, subject to different pIRE treatment plans with PS-OFDI. Burn injuries in rats have obvious limitations in modeling the healing of burn wounds in humans (Ramos et al., 2008); yet such animal studies (Mitsunaga et al., 2012) enable the development and investigation of PS-OFDI signatures *in vivo* during wound healing and are an essential step toward diagnostic applications in human patients (Lo et al., 2016).

In this study, we showed that the scar size 6 months after third-degree burn injury in rats can be accurately estimated from non-invasive measurements with PS-OFDI performed in the first 3 months after the injury. The best results (Pearson coefficient 0.57, *p* < 10^−4^ and Spearman coefficient 0.66, *p* < 10^−5^, Figure 4C) was achieved when predicting the scar area at month 6 from the scar area, the birefringence homogeneity and the slope of the DOP measured 2 and 3 months after the injury.

The results of this study point to the potential of developing an approach for predicting clinically important scar properties early in the treatment process. The relevance and scope of the current results are, however, limited. Our results are clearly valid in the context of scars in rats and under the protocols used herein. It is likely that models can be developed for human scars, of many possible types, but the parameters presented are clearly not directly transferable. Even in the context of rats models, a major limitation of this current study is the limited number of tested samples. While the sample size is sufficient to support a strong confidence level that the prediction quality is not spurious larger data sets, with hundreds of independent scars, are required to construct and test more precise prediction models. Such larger samples will allow for more rigorous statistical testing, replacing the LOOCV approach used here. The sample size limitation also dictated a composition of our analyzed cohort that is composed of untreated samples as well as from several different treatments. A more uniform design can lead to even more accurate results but could not be used here as the sample set would become too small.

The predicted quantity at month 6 clearly depends on the rat's month 3 to month 6 history which is not captured by the month 1 to month 3 measurements. Prediction accuracy is also affected by the measurement accuracy. Scar size was manually measured and therefore, the measurement depends on the evaluator. Automated assessment of the scar size, for example using OCT, would be favorable and remove bias, however this method was not performed in the present study as the method will limit the observed field of view. In addition, genetic variation between the rats in this study could have affected scarring potential but are not yet captured in our model. Larger studies will not be able to resolve prediction errors due to these factors. They can allow more complex models, however, and the inclusion of more factors, such as quantitative histological features information (Quinn et al., 2014). Since different signals would likely be detected in human wounds in comparison to wounds in rats, as indicated above, larger future studies should also address differences and how model parameters can translate to human scars. Our analysis and modeling methodology can be extended to perform larger studies in different contexts.

## Author contributions

EK and ZY developed the prediction model and drafted the manuscript, MV and BB conducted the PS-OFDI analysis, EK, ZY, MV, MY, AG drafted the manuscript, AG conceived the study and performed data analysis. All authors read and edited the manuscript.

### Conflict of interest statement

The authors declare that the research was conducted in the absence of any commercial or financial relationships that could be construed as a potential conflict of interest.
