# Supplementary material for: Prediction of Scar Size in Rats Six Months after Burns Based on Early Post-injury Polarization-Sensitive Optical Frequency Domain Imaging
Source: Front Physiol. 2017 Dec 1;8:967. doi: 10.3389/fphys.2017.00967 (PMC5717021; doi:10.3389/fphys.2017.00967)
Supplement: Supplementary file 1 [file DataSheet1.pdf]

Supplementary information

Table S1. Parameters of pulsed electric fields applied to burn wounds to modify scar formation as described in ref[23]. 3 repeats per each wound.

Voltage (V)	Number of pulses (N)	Treatment frequency (days)	Scar_Area	Hom	DOPSlope
Normal skin			100	100	100
Untreated burn			0	0	0
125	200	10	16	14	26
125	400	20	17	29	19
125	800	30	19	14	16
250	200	20	58	57	37
250	400	30	24	14	23
250	800	10	50	14	26
500	200	30	26	29	33
500	400	10	29	-29	19
500	800	20	32	-21	26

Table S2. Coefficients of the linear model based on OFDI measurements at Month 1. (n=36 wounds, 9 animals, OFDI data acquired from 2-3 volumes from each wound).

	Scar Area (α_1)	Hom (γ_1)	DOPSlope (δ_1)	Intercept (β_0)
count	36	36	36	36
mean	-2.783	0.643	-2.171	40.314
std	0.288	1.289	0.647	1.392
min	-3.657	-2.574	-3.679	34.281
25%	-2.899	0.350	-2.281	40.078
50%	-2.795	0.591	-2.175	40.319
75%	-2.635	0.903	-2.027	40.606
max	-2.144	6.642	0.865	43.869
std/mean	-0.104	2.003	-0.298	0.035

Table S3. Coefficients of the linear model based on OFDI measurements at Month 2. (n=36 wounds, 9 animals, OFDI data acquired from 2-3 volumes from each wound).

	Scar Area (α_2)	Hom (γ_2)	DOPSlope (δ_2)	Intercept (β_0)
count	36	36	36	36
mean	10.880	-8.524	-5.258	36.85
std	0.8654	1.289	0.514	0.779
min	9.779	-9.953	-7.287	34.256
25%	10.472	-9.009	-5.445	36.674
50%	10.853	-8.754	-5.268	36.904

75%	10.926	-8.525	-5.016	37.153
max	15.213	-2.273	-3.908	38.395
std/mean	0.079	-0.151	-0.098	0.021

Table S4. Coefficients of the linear model based on OFDI measurements at Month 3. (n=36 wounds, 9 animals, OFDI data acquired from 2-3 volumes from each wound).

	Scar Area (α_3)	Hom (γ_3)	DOPSlope (δ_3)	Intercept (β_0)
count	36	36	36	36
mean	12.197	-0.502	-4.994	40.165
std	0.896	0.356	0.316	0.710
min	10.045	-1.952	-5.637	38.282
25%	11.888	-0.581	-5.109	39.924
50%	12.313	-0.497	-4.997	40.178
75%	12.611	-0.359	-4.846	40.339
max	14.230	0.739	-4.098	42.272
std/mean	0.073	-0.709	-0.063	0.018

Table S5. Coefficients of the linear model based on OFDI measurements at Months.

1&2. (n=36 wounds, 9 animals, OFDI data acquired from 2-3 volumes from each wound).

	ScarArea (α_1)	Hom (γ_1)	DOPSlope (δ_1)	Scar Area (α_2)	Hom (γ_2)	DOPSlope (δ_2)	Intercept (β_0)
count	36	36	36	36	36	36	36
mean	-1.930	-2.971	-3.2	11.028	-9.014	-5.149	43.556
std	0.276	1.321	0.643	0.750	1.301	0.554	1.413
min	-2.684	-6.755	-5.509	9.743	-10.694	-6.738	38.083
25%	-2.030	-3.096	-3.292	10.558	-9.659	-5.403	43.370
50%	-1.914	-2.950	-3.204	10.972	-9.263	-5.115	43.614
75%	-1.774	-2.607	-3.090	11.286	-8.736	-4.914	43.887
max	-1.206	1.861	-0.554	14.275	-3.983	-3.839	48.359

Table S6. Coefficients of the linear model based on OFDI measurements at Months.

1&3. (n=36 wounds, 9 animals, OFDI data acquired from 2-3 volumes from each wound).

	Scar Area (α_1)	Hom (γ_1)	DOPSlope (δ_1)	Scar Area (α_3)	Hom (γ_3)	DOPSlope (δ_3)	Intercept (β_0)
count	36	36	36	36	36	36	36
mean	-1.969	2.562	0.953	11.521	-1.183	-4.415	40.168
std	0.236	1.006	0.442	1.08	0.403	0.359	1.033
min	-2.585	-1.185	-0.585	7.896	-2.733	-5.433	37.682
25%	-2.109	2.438	0.816	11.198	-1.335	-4.543	39.771
50%	-1.952	2.676	0.967	11.642	-1.213	-4.361	40.120
75%	-1.850	2.943	1.048	11.875	-1.078	-4.203	40.527
max	-1.551	5.187	2.438	13.937	-0.126	-3.767	43.695

std/mean	-0.120	0.391	0.463	0.094	-0.341	-0.081	0.026

Table S7. Coefficients of the linear model based on OFDI measurements at Months.

2&3. (n=36 wounds, 9 animals, OFDI data acquired from 2-3 volumes from each wound).

	**Scar Area (α_2)**	**Hom (γ_2)**	**DOPSlope (δ_2)**	**Scar Area (α_3)**	**Hom (γ_3)**	**DOPSlope (δ_3)**	**Intercept (β_0)**
count	36	36	36	36	36	36	36
mean	7.279	-14.027	-4.564	10.589	0.574	-6.210	43.812
std	0.659	1.267	0.435	0.987	0.442	0.525	1.094
min	5.866	-16.993	-5.474	8.586	-1.380	-7.824	41.305
25%	6.978	-14.326	-4.816	10.102	0.487	-6.436	43.345
50%	7.236	-14.079	-4.603	10.559	0.591	-6.249	43.754
75%	7.399	-13.688	-4.274	10.959	0.684	-6.035	43.989
max	9.694	-10.555	-3.576	13.349	1.524	-4.938	47.799
std/mean	0.091	-0.090	-0.095	0.093	0.770	-0.085	0.025

Table S8. Coefficients of the linear model based on OFDI measurements at Months 1&2&3. (n=36 wounds, 9 animals, OFDI data acquired from 2-3 volumes from each wound).

	**Scar Area (α_1)**	**Hom (γ_1)**	**DOPSlope (δ_1)**	**Scar Area (α_2)**	**Hom (γ_2)**	**DOPSlope (δ_2)**	**Scar Area (α_3)**	**Hom (γ_3)**	**DOPSlope (δ_3)**	**Intercept (β_0)**
count	36	36	36	36	36	36	36	36	36	36
mean	-0.045	-3.798	-6.085	0.352	-100.55	-46.253	0.4477	2.530	-58.548	101.655
std	0.012	8.045	4.896	0.032	11.141	5.462	0.051	3.639	6.462	10.396
min	-0.072	-23.849	-24.155	0.281	-127.903	-56.609	0.3353	-12.716	-77.475	70.225
25%	-0.049	-4.823	-7.940	0.338	-103.975	-50.212	0.4255	1.680	-60.369	97.430
50%	-0.044	-2.249	-5.542	0.350	-100.996	-46.759	0.4511	2.516	-58.400	102.425
75%	-0.040	-0.480	-4.313	0.364	-97.171	-43.109	0.4671	3.623	-56.162	105.164
max	-0.012	14.563	7.813	0.459	-73.719	-34.232	0.5866	10.683	-43.930	127.364
